# Comparative Efficiency of Whole-Body Electromyostimulation and Resistance Training in Enhancing 1-Repetition Maximum

**DOI:** 10.3390/jfmk10030243

**Published:** 2025-06-26

**Authors:** Valentina Grgic, Ludovico Grossio, Anna Mulasso, Gennaro Boccia, Alberto Rainoldi

**Affiliations:** 1 Department of Clinical and Biological Sciences, University of Turin, 10126 Turin, Italy; valentina.grgic@unito.it; 2 Neuromuscular Function Research Group, School of Exercise and Sport Science, University of Turin, 10126 Turin, Italy; ludovico.grossio@unito.it (L.G.); anna.mulasso@unito.it (A.M.); alberto.rainoldi@unito.it (A.R.); 3 National PhD Programme in Kinesiology and Sport Sciences, University of Verona, 37129 Verona, Italy; 4 Department of Medical Sciences, University of Turin, 10126 Turin, Italy

**Keywords:** WB-EMS, resistance training, women, back squat, hammer curl, plank

## Abstract

**Background**: Whole-body electromyostimulation (WB-EMS) combines full-body electrical muscle stimulation with instructor-assigned exercise. Electrical impulses are transmitted to the peripheral muscles through electrodes applied to the body. This study compared two training methodologies, WB-EMS training and traditional resistance training, to determine which approach leads to greater strength improvement in terms of 1-repetition maximum (1-RM). **Methods**: Twenty sedentary women participated in a 10 weeks protocol with five evaluations conducted every two weeks. The WB-EMS group trained for 20 min per week, and the resistance training group (RT) performed an average of two training sessions per week, lasting 60 min each. Both groups were evaluated using three exercises: back squat and hammer curl (1-RM), and plank exercise (time to exhaustion). **Results**: Both groups increased their performance in squat (WB-EMS +36%, *p* = 0.0001; RT +34%, *p* = 0.0001), curl (WB-EMS +42%, *p* = 0.0001; RT +33%, *p* = 0.0001), and plank (WB-EMS +103%, *p* = 0.0001; RT +65%, *p* = 0.0001). No significant time × training interaction was found for any exercise, indicating that the two groups improved similarly. **Conclusions**: Although WB-EMS did not confer greater strength improvement than traditional resistance training, it offers a time-efficient alternative, achieving similar results with reduced time commitment.

## 1. Introduction

Whole-body electromyostimulation (WB-EMS) is an innovative training method designed to enhance muscle strength and mass. It involves the voluntary simultaneous co-activation of agonist and antagonist muscle groups while performing bodyweight exercises or using light external loads. This approach ensures balanced and comprehensive engagement of multiple muscle groups. Electrodes are strategically placed on the superficial muscles of the trunk, hips, arms, and thighs, delivering electrical impulses to the peripheral nerve endings. These impulses induce superimposed evoked contractions, effectively enhancing the intensity of muscular engagement beyond that typically achievable through voluntary co-contraction alone. This combination of voluntary effort and externally induced contractions creates an effective training stimulus [1]. Standard training protocols for WB-EMS typically involve specific stimulation parameters: frequency of 85 Hz, impulse duration of 4 s, pause duration of 4 s, and square wave duration of 350 µs. Sessions are relatively brief, lasting around 20 min, and are generally conducted once a week, making WB-EMS a time-efficient option for improving strength and conditioning.

A number of studies on the effects of WB-EMS are now available in the recent literature. WB-EMS is described as a method to improve body composition [2] and reshape physical fitness [3], increasing the quality and quantity of lean mass [4], decreasing fat mass, and inducing metabolic changes [5]. This method is particularly popular among individuals seeking to improve muscle strength, endurance, and overall physical fitness with minimal joint strain compared to traditional high-load resistance training. WB-EMS training is suitable for virtually everyone, especially those who are unable to lift external loads due to back pain [6,7], joint problems, sarcopenia [8,9], and osteopenia [10,11]. This training method has also been adopted in the world of fitness [12] and athletic preparation [13,14,15]. There are some absolute contraindications, such as pregnancy, cardiac pacemakers or electrical implants, and epilepsy [16]. Furthermore, electrical stimulation training combined with eccentric contractions can cause rhabdomyolysis [17,18].

This improvement in muscle strength is the most investigated variable in the WB-EMS training literature. In 2017, it was demonstrated that 15 min of WB-EMS training induced a higher energy expenditure than 15 min of traditional resistance exercise [19]. Qin et al. indicate that WB-EMS training can lead to an 18–23% increase in biceps strength over six weeks, significantly surpassing the 8–12% increase observed with traditional training [20]. Furthermore, an eight-week WB-EMS training program resulted in a 7.7% increase in maximal isometric strength in the lower limbs, compared to a 2.1% increase in a group following a comparable traditional training protocol [21]. Finally, Kemmler et al. demonstrated that 16 weeks of WB-EMS training can increase quadriceps strength by 7.3 ± 10.3%, compared to 12.7 ± 14.7% in the HIIT training group [22]. There is a notable lack of studies directly comparing WB-EMS with traditional training methods. The few studies that have compared these methods used a wide variety of training protocols, ranging from one session per week [23] to five sessions per week; in contrast, others differ in terms of frequency (85 or 7 Hz) and duration of the impulse (6 or 4 s). These protocol variabilities complicate the ability to draw clear and consistent conclusions regarding the relative effectiveness of WB-EMS with respect to traditional training. This lack of knowledge is particularly critical, limiting the evidence-based choice of the best intervention, mainly in the world of rehabilitation and adapted physical activity in the choice of best intervention. If WB-EMS training produces the same effects as resistance training, a time-effective analysis can be performed.

Consequently, the aim of the study was to compare two training methodologies, WB-EMS training and traditional resistance training (RT, active control group), in terms of strength improvement in a group of 20 women. We consider it important to analyze the effects of WB-EMS training on young, healthy women, as the existing scientific literature primarily focuses on older women or those in post-menopause [2,3]. We compared strength gains measured as an improvement in 1-RM on back squat and biceps hammer curl, or time to exhaustion in a plank exercise over a 10-week period. In our hypothesis, the results of both groups would be similar in terms of relative strength increase.

## 2. Materials and Methods

### 2.1. Participants

A convenient sample of 20 women was screened for participation. The inclusion criteria were a sedentary lifestyle for at least six months and a body mass index (BMI) within the normal range (18–25 kg/m^2^). The exclusion criteria were the presence of musculoskeletal complaints or smoking. All participants met the inclusion criteria and did not meet any exclusion criteria; therefore, no individuals were excluded during the screening process. The average age of the sample was 20 ± 3 years. All participants were informed about the testing procedure and provided written informed consent prior to their participation in this study, which was approved by the local Ethical Advisory Committee and performed in accordance with the Declaration of Helsinki. We recommend not consuming caffeine, theophylline, or any stimulating substances in the hours leading up to the tests, as these could influence the test results. Furthermore, participants were instructed to avoid intense physical exercise the day before the evaluation to prevent altered results due to delayed-onset muscle soreness (DOMS). The allocation was not randomized but was intentional, based on each participant’s choice of preferred training modality.

### 2.2. Experimental Design

It is important to recognize that traditional resistance training and WB-EMS are fundamentally different, making direct comparison challenging. Specifically, WB-EMS involves unique contraction patterns, primarily isometric contractions, distinct timing dictated by electrical impulses, and a limited range of motion. The execution of WB-EMS exercises is inherently tied to electrical stimulation; for example, WB-EMS sessions are typically performed without external overload, whereas traditional resistance training without added load would be ineffective. This means that it is not feasible to standardize exercise execution across both modalities while isolating only the presence or absence of electrical stimulation. Given these differences, our comparison relied on their respective international guidelines, which reflect the specific exercises and equipment typically used in each training method rather than identical protocols. The study consisted of 10 weeks of training, with assessments conducted every two weeks (weeks 2, 4, 6, 8, and 10), for a total of 5 assessment sessions. The measurements were taken every two weeks, and the 1 RM was assessed for back squat and hammer curl, and the time to exhaustion was assessed for the plank. The participants were assigned to the two groups based only on their intentions and personal choices. The 20 women were divided into two groups: 10 in the WB-EMS group and 10 in the resistance training (RT) group. Each participant was trained separately, and each session was supervised by a personal trainer.

### 2.3. Training

The women in the experimental group engaged in one session of WB-EMS training per week for 20 min. Throughout the study, the participants followed the advanced program of the Mihabodytec device (Mihabodytec^®^, Gersthofen, Germany). Electrodes were applied to the biceps, pectoralis, abdominals, shoulders, dorsalis, lower back, glutes, and quadriceps, and the parameters were set according to the international electrostimulation guidelines [24]. The parameters were set as follows: frequency, 85 Hz; impulse duration, 4 s; pause duration, 4 s; and amplitude, 350 µs. Each exercise was performed in a single set of 10–12 repetitions with a 4 s pause. The eight exercises most frequently performed were squats, lunges, kickbacks, planks, standing bicycles, hollow positions, curls, and pushdowns (details are reported in Table 1).

In contrast, participants in the control group (RT) performed an average of 2 ± 1 training sessions per week, each lasting approximately 60 to 90 min. They followed a strength training program using traditional gym equipment: 5 to 8 exercises per session, focusing on the upper limbs, core, and lower limbs, with 1 to 3 sets of 8 to 20 repetitions each, with 1 min of rest between sets. In the RT group, exercises similar to those in the WB-EMS group were used in order to recruit the same muscle groups as much as possible. The exercises most frequently performed were squats, lunges, leg extensions, planks, crunches, curls, pushdowns (details are reported in Table 1).

### 2.4. Assessment

Both groups were evaluated on three exercises to monitor performance variations throughout the study:

- Isometric Plank: this exercise involves maintaining a static position with the body parallel to the ground, supported on the elbows, forearms, and toes (until time to exhaustion).
- Back Squat: begins with a barbell positioned behind the shoulders; participants perform a squat, bringing the hips down to knee level, and then return to the starting position (1-RM).
- Hammer Curl: involves actively flexing the elbow with a “hammer” grip, starting with arms at the sides and bringing the dumbbells close to the shoulders (1-RM).

The isometric plank was performed as the first assessment, and the time until exhaustion was recorded. For the other two exercises, the participants were asked to lift the load as many times as possible. The load was individually chosen by the experimenter in order to allow for 4 to 10 repetitions. The one-repetition maximum (1-RM) was calculated using Baechle’s [25] formula provided here: 1-RM = weight lifted (kg) × (1 + (maximum number of repetitions × 0.033))

### 2.5. Statistical Analysis

After checking for normality using the Shapiro–Wilk test, the data are presented as mean standard deviation (SD). To compare the results obtained by each group for each exercise, a two-way repeated-measures ANOVA (time × training) was used. The level of statistical significance was set at *p* = 0.05, and a Bonferroni post-hoc test correction was performed for each comparison. The magnitude of the effect size (Cohen’s d) was defined as “trivial” < 0.2, “small” ≤ 0.5, “moderate” ≤ 0.8, large ≤ 1.4, and very large ≥ 1.4. Statistical analyses were performed with JASP (version 0.19.1.0).

## 3. Results

All women underwent testing according to the protocol. No injuries or undesired side effects occurred during the 10 weeks. The results of the Shapiro-Wilk test confirmed a normal distribution for the three WB-EMS exercises, except for the back squat in the first 4 weeks, the plank in the 6th week, the hammer curl in the first two weeks, and the last two weeks.

All descriptive values are presented in Table 1 and Table 2, Supplementary Material reports the raw data for each subject. Repeated measures ANOVA revealed a significant effect of time, while no significant effect of training was found. The post-hoc results are presented in Table 2.

**Figure 1:**
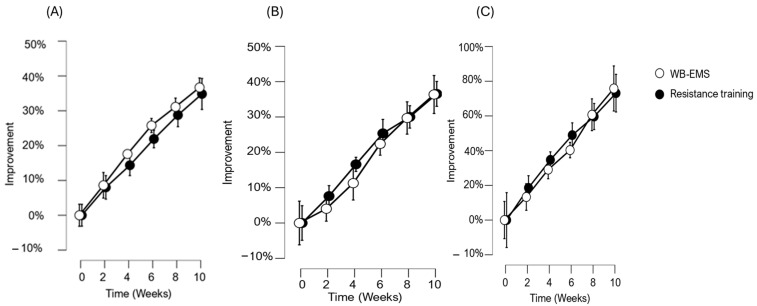
Percentage trends of improvement over a 10 weeks protocol of (**A**) back squat, (**B**) hammer curl, and (**C**) plank.

1. **Back squat**: ANOVA showed a main effect of time (*p* < 0.001) without a significant interaction of time × group (*p* < 0.685). In week 0, the WB-EMS group achieved an average of 47.0 ± 4.7 kg, which significantly improved to 64.0 ± 5.0 kg by the end of the protocol. Similarly, the resistance training group started with an average of 52.7 ± 8.5 kg in week 0 and increased their performance to 70.4 ± 11.5 kg by week 10. In addition, post hoc analysis indicated a considerable effect size (Cohen’s d = 2.171, *p* < 0.001). (Figure 1).
2. **Hammer Curl**: ANOVA showed a main effect of time (*p* < 0.001) without a significant interaction time × group (*p* < 0.602). In week 0, the WB-EMS group achieved an average of 7.3 ± 2.6 kg, which significantly improved to 10.0 ± 3.5 kg by the end of the study. Similarly, the resistance training group started with an average of 12.4 ± 5.5 kg in week 0 and increased their performance to 16.6 ± 6.5 kg by week 10. Post hoc analysis indicated a moderate effect size (Cohen’s d = 0.713, *p* < 0.001). (Figure 1).
3. **Plank**: ANOVA showed a main effect of time (*p* < 0.001) without a significant interaction time × group (*p* < 0.694). In week 0, the WB-EMS group held the plank for an average of 79.4 ± 19.1 s, which improved significantly to 161.2 ± 57.8 s by the end of the protocol. Similarly, the resistance training group increased from an average of 97.1 ± 39.5 s in week 0 to 161.2 ± 57.8 s by week 10. Post-hoc analysis indicated a substantial effect size (Cohen’s d = 1.529, *p* < 0.001). (Figure 1).

## 4. Discussion

The aim of this study was to compare the effects of the two training methods on strength improvement. However, it is important to highlight that the two methods are significantly different, making a direct comparison impossible. Consequently, we analyzed and compared them based on their respective international guidelines. The WB-EMS group trained for 20 min per week using a Mihabodytec device (frequency, 85 Hz; impulse duration, 4 s; pause duration, 4 s; amplitude, 350 µs). The resistance training group performed an average of 2 ± 1 training sessions per week, each lasting approximately 60 to 90 min. The protocol included 10 weeks of training, with a 1-RM test (back squat and hammer curl) and time to exhaustion (plank) evaluated every 2 weeks.

Both groups showed similar improvements in their performance for each exercise. In fact, a significant effect of time and a non-significant effect of training were found in the repeated-measures ANOVA. The back squat in the WB-EMS training group showed substantial significant differences in the five measurements, following a consistent linear trend (*p* > 0.005). These results are consistent with existing research: Micke et al. showed that two WB-EMS training sessions per week can improve leg strength and power [21], and Ludwig et al. showed that a 10-week WB-EMS training program can improve knee extensor strength in a football population [26]. Although the parameters used were different from those in our study, Filipovic et al. demonstrated that two WB-EMS sessions per week increased lower limb strength, as measured by 1-RM on the leg extension exercise [27]. While the back squat assessments showed excellent results, exceeding the expected target, the hammer curl results were more modest. These results are consistent with the literature: Qin et al. compared biceps strength gains between the WB-EMS and resistance training groups, and the improvements over 6 weeks were (18 ± 23 kg) and (8 ± 17 kg), respectively [20]. Pichon demonstrated that WB-EMS training can enhance upper limb strength in swimmers [28]; similarly, Evangelista showed that inactive older men can increase arm curl strength through WB-EMS combined with weight training [29].

For the isometric plank, a very rapid initial rate of improvement was observed in both groups, suggesting a faster adaptive muscular response during early assessments. The trend in performance was also observed for this exercise, with a *p*-value < 0.005, again indicating significant differences across the five assessments. These results starkly contrast with those of the existing literature. Amaro-Gahete et al. assessed changes in core strength in sedentary adults over 12 weeks. They found a significantly greater improvement in plank performance in the WB-EMS group (+64% at the end of the protocol) [30]. Similarly, Zink-Rückel et al. demonstrated that WB-EMS can improve core stability (+5–7% in 16 weeks) in amateur golfers, but their test focused on strength (Maximum Isometric Trunk Strength, MITS), whereas we assessed time to exhaustion during the plank exercise [15]. In addition, Shalamzari et al. showed that WB-EMS training improved core stability in individuals with lumbar lordosis [31].

One limitation of this study is the inability to conduct a direct comparison between the two training methods. Additionally, the small number of female participants (in line with other studies) and the lack of randomization limit the generalizability of these findings. Another notable limitation is the absence of a crossover design, which would have allowed for within-subject comparisons. Finally, the fact that participants self-selected their preferred training modality may have influenced the outcomes due to potential bias or differences in motivation and adherence to the training program. Additionally, dietary control was not implemented because the participants were amateurs. This factor may have influenced the results. Future research could address these limitations by focusing on different target populations, such as athletes, older adults, or individuals of different sexes. It could also involve testing different exercises or incorporating other variables to better understand the effects of training methods.

## 5. Conclusions

These findings suggest that WB-EMS training is as effective as traditional resistance training. While the two training modalities are equally effective, WB-EMS is more efficient as it requires only 20 min a week instead of two hours. WB-EMS appears to be more efficient than conventional gym workouts for young, sedentary women, as it produces similar results while requiring a significantly smaller time investment from participants. Our initial hypothesis that WB-EMS and resistance training would yield comparable strength improvements was confirmed.

## Figures and Tables

**Table 1:** Example of a session training for both methods.

	WB-EMS	Resistance Training (RT)
**Warm-up**	mobility	cardio and mobility
**Lower body exercises**	squats and lungs (with variations), leg kicks	squats, lunges, leg press, leg extensions, leg curls, and variations
**Core exercises**	standing bicycle, standing crunches, planks, hollow position	crunches, planks, mountain climbers, Pallof press, and variations
**Upper body exercises**	curls and variations, triceps extensions, TRX rows, and presses	curls and variations, shoulder and chest presses, triceps pushdowns, lateral raises, and variations
**Cool-down**	body relax program	stretching

**Table 2:** Post Hoc test and Cohen’s d effect size on data performance improvements.

Back Squat						
		**Mean Difference**	**SE**	**t**	**Cohen’s d**	***p*** **_bonf_**
Week 0	Week 2	4.30	0.57	7.49	0.53	<0.001
	Week 4	7.75	0.53	14.38	0.97	<0.001
	Week 6	11.70	0.70	16.66	1.46	<0.001
	Week 8	14.85	0.76	19.43	1.85	<0.001
	Week 10	17.35	0.69	24.80	2.10	<0.001
**Hammer curl**						
Week 0	Week 2	0.57	0.11	4.96	−0.11	0.002
	Week 4	1.30	0.17	7.57	0.27	<0.001
	Week 6	2.29	0.26	8.76	0.47	<0.001
	Week 8	2.86	0.29	9.81	0.59	<0.001
	Week 10	3.43	0.28	11.97	0.71	<0.001
**Plank**						
Week 0	Week 2	13.40	2.91	4.59	0.34	0.003
	Week 4	26.80	3.71	7.21	0.68	<0.001
	Week 6	37.30	4.30	8.65	0.95	<0.001
	Week 8	49.10	4.85	10.11	1.25	<0.001
	Week 10	59.95	4.94	12.13	1.52	<0.001

Note. *p*-value adjusted for comparing a family of 15. The results are averaged over the levels of TRAINING.

## Data Availability

Data used in this study may be shared upon reasonable request.

## Supplementary Materials

The following supporting information can be downloaded at: https://www.mdpi.com/article/10.3390/jfmk10030243/s1.

## Abbreviations

The following abbreviations are used in this manuscript:

ANOVA	Analysis of variance
WB-EMS	Whole-body electromyostimulation
RT	Resistance training
BMI	Body Mass Index
SD	Standard deviation
MVC	Maximum voluntary contraction
